# An exploratory study to investigate the effects of motor performance and maturation on talent selection in handball

**DOI:** 10.3389/fpsyg.2026.1773319

**Published:** 2026-04-28

**Authors:** Leon Brüning, Christian Winter, Fabienne Döringer, Mark Pfeiffer

**Affiliations:** Theory and Practice of Sports, Department 02 Social Sciences, Media and Sport, Johannes Gutenberg-University, Mainz, Germany

**Keywords:** talent identification, coaches, decision making, person-centered, variable-centered

## Abstract

Talent selection, as an important component of talent development, is often examined in the literature using a variable- or person-centered approach in order to better understand which criteria are used in the selection decision. The present study compares the two approaches to investigate the link between motor performance and nomination status in youth. This included both the selection decision at state level (talent selection I) and the nomination for the national team trials (talent selection II). A sample of 331 male (*M*_*Age*_ = 12.96 ± 0.19) and 348 female (*M*_*Age*_ = 12.92 ± 0.29) handball players completed six motor performance tests (20-m Sprint, Jump and Reach, Shuttle Run, Push-Up Test, Throwing Velocity, Dribbling Test) as part of the state squad selection process. To measure maturation, anthropometric values of the athletes were collected and the Mirwald equation was used (Mirwald et al., 2002). To analyze the link between individual test results (independent variables) and squad nomination (dichotomous dependent variable), a logistic regression model (*Nagelkerke*′*s R*^2^, Odds Ratio) was used, differentiated by gender and squad nomination level (variable-centered). In addition, a cluster analysis (Ward's method, *k*-means) was performed using the person-centered approach. The relationship between the identified clusters and the nomination of executives was examined (Odds Ratio). Logistic regression showed that throwing performance had a significant influence on talent selection I for both boys and girls (*OR*_*Boys*_ = 1.8, *p* < 0.05; *OR*_*Girls*_ = 2.73, *p* < 0.05). For girls, a significant influence on talent selection II (*OR* = 1.93, *p* < 0.05) was also found. Based on the cluster analysis, four clusters were identified for boys and five clusters for girls. Across genders, athletes in one cluster are significantly more likely to be nominated for the State-Squad (*OR*_*Boys*_ = 3.71, *p* < 0.05; *OR*_*Girls*_ = 3.67, *p* < 0.05). This cluster is characterized by above-average performance in all tests. In the case of girls, this link can also be demonstrated in talent selection II (*OR* = 2.66, *p* < 0.05). The higher effect sizes of the person-centered approach compared to the variable-centered approach argue in favor of a more holistic view of motor skills in sport in the form of a profile analysis for talent selection.

## 1 Introduction

Financial support for sports associations is largely linked to their sporting success. A key criterion for the allocation of funds is success at major international events such as the Olympic Games and World Championships. Therefore, a key objective of sports associations is to identify talented athletes at an early stage to provide them with the best possible support on their way to major sporting events. The aim is to identify athletes who have the potential to perform at the highest level as adults (Faber et al., 2023; Vaeyens et al., 2009). Due to the limited human, financial and structural resources of the professional associations, support is limited to a select group of particularly promising young athletes (Hohmann and Siener, 2021). In sport science literature, this process is examined under the concept of Talent Identification and Development (TID). TID is not a one-off event, but a multi-stage process. This process can be divided into successive phases: talent detection, talent identification, talent selection and talent development (Baker et al., 2017).

As key actors in the talent selection phase, sports associations bear primary responsibility for organizing talent selection activities within their development frameworks, such as through selection camps. This multi-year process entails a sequence of successive selection decisions, during which athletes' potential is continuously evaluated—for instance, through progression from regional to national squads. In parallel, talent selection also occurs at the club level (Leite et al., 2021), where decisions are made regarding athletes' advancement to higher age-group or performance teams. These club-level selections determine which athletes remain embedded in the internal talent development pathway of the club, thereby complementing and contextualizing the broader selection processes administered by associations.

The stepwise nature of talent selection is largely attributable to the inherent prognostic uncertainty in predicting long-term athletic success based on early performance indicators at the onset of sport-specific, performance-oriented training (Güllich and Emrich, 2014). This uncertainty arises from the complex, multifactorial nature of performance, wherein performance emerges from the dynamic interplay of physiological, psychological, technical-tactical, and environmental factors (Elferink-Gemser et al., 2007; Hohmann, 2009; Williams and Reilly, 2000). Furthermore, peak performance cannot be attributed to a standard set of talent criteria. Rather, individual combinations of talent criteria can lead to peak performance (Williams and Reilly, 2000). Due to this complex and multifactorial composition of athletic performance, successive talent selection decisions in sports practice represent a repeated validation of talent assessment and thus a prognosis at shorter intervals (Schorer et al., 2017).

Empirical studies examining the prognostic validity of talent selection do not sufficiently differentiate with regard to the criterion for operationalizing talent. On the one hand, athletic performance in adulthood (e.g., reaching the professional league) is used as a criterion (e.g., Höner et al., 2017; Johnston et al., 2018; Leyhr et al., 2018; Schorer et al., 2017, 2020). On the other hand, the squad status achieved in the promotion system (e.g., national squad) is found in studies as a criterion (Johnston et al., 2018; Zibung et al., 2016; Zibung and Conzelmann, 2013; Zuber et al., 2014, 2016). While using squad status as a talent criterion does not allow any conclusions to be drawn about the quality of the selection in terms of performance in adulthood, it does provide a better understanding of the talent characteristics on which the selection decision is based (Baker et al., 2017; Johnston et al., 2018; Nijenhuis et al., 2024; Till and Baker, 2020). Since selection decisions are linked to access to further support structures and thus have a strong influence on the further development of athletes, it is of interest to examine the selection criteria in greater depth. Studies examining selection criteria frequently rely on motor performance tests, as motor skills constitute a fundamental basis of athletic performance (Weigel, 2014). These abilities encompass several performance dimensions: endurance, strength, speed, and flexibility. However, current research considers motor performance in childhood within the broader context of biological maturation, recognizing its substantial impact on children's performance (Aouichaoui et al., 2024; La Rubia et al., 2024). Biological maturation comprises multiple subsystems, including hormonal processes, the development of secondary sexual characteristics, skeletal age, and individual growth patterns. Within talent selection contexts, growth is often emphasized due to its high practical applicability and is commonly operationalized through the assessment of the age of peak high velocity (PHV). PHV can be determined using invasive methods (e.g., hand–wrist radiographs) as well as non-invasive estimation procedures based on anthropometric measures. Owing to their logistical advantages, non-invasive approaches are predominantly used in applied sport settings (Mirwald et al., 2002). Accordingly, studies investigating the relationship between PHV, motor performance and talent selection decisions frequently employ the maturity offset prediction equation proposed by Mirwald et al. (2002), Abbott et al. (2025), Aouichaoui et al. (2024) and Thieschäfer et al. (2025).

With regard to research design, studies on motor performance and maturation in the context of talent selection can be grouped into two different research approaches. These approaches differ in their methodological focus and analytical perspective and are outlined in the following section.

Numerous studies use a variable approach to examine the relationship between juvenile performance and squad selection (Elferink-Gemser et al., 2004, 2007; Huijgen et al., 2014; Lidor et al., 2009; Phillips et al., 2010; Schorer et al., 2017; Woods et al., 2016). According to this approach, individual talent characteristics are examined with regard to squad selection. Nevertheless, current research findings on talent selection in sports, particularly in handball, allow only limited conclusions to be drawn about the explanatory power of individual measurement instruments on selection decisions (Schorer et al., 2017; Till and Baker, 2020). The limited explanatory power in previous studies can be attributed to various content-related and methodological causes. One possible cause may be that the variable-oriented approach, by considering individual talent characteristics in isolation, fails to take into account interactions between the various characteristics (Baker et al., 2017).

In contrast, the person-centered approach focuses on the interactions among multiple factors, rather than examining individual variables in isolation (Howard and Hoffman, 2018; Morin et al., 2018; Saqr et al., 2024). This research approach forms subpopulations from a population. These subpopulations represent complex profiles based on several variables. In the context of talent identification, a person-centered approach enables the clustering of athletes into distinct profiles based on selected talent-related characteristics. These profiles can then be examined in relation to a specific outcome variable—such as the nomination status—in order to uncover correlations between certain characteristic constellations and sporting success. Initial studies on the person-centered approach examine characteristics relating to training history as well as psychological and motor skills characteristics (Schmid et al., 2020, 2021; Zibung et al., 2016; Zibung and Conzelmann, 2013; Zuber et al., 2014, 2016). Since examining talent criteria in their entirety is extremely complex in terms of research methodology (economics), the focus is often limited to subsystem. This makes it possible to describe the subsystem in greater depth (Zibung et al., 2016; Zuber et al., 2016).

Studies adopting both person-centered and variable-centered approaches investigating motor performance and maturation with regard to talent selection in handball are already available. However, these studies often differ in terms of research approaches, measurement methods used, statistical procedures and samples (La Rubia et al., 2024; Matthys et al., 2011; Schorer et al., 2017; Tróznai et al., 2024). This makes it difficult to arrive at a uniform assessment of relevant talent criteria. This study is therefore exploratory in nature and pursues the following two objectives:

- To investigate the influence of motor performance and maturity on selection decisions at different stages of talent selection process according to two research approaches (variable-centered and person-centered),

More specifically, the variable-centered approach examines the influence of performance abilities measured by six motor skill tests and PHV on squad selection. The person-centered approach derives profiles based on these individual characteristics to analyze which profile configurations are associated with particularly favorable selection prospects.

- To compare the two research approaches with regard to the explanatory power of selection decisions at different stages of the talent selection process.

## 2 Materials and methods

### 2.1 The selection process

**Figure 1:**
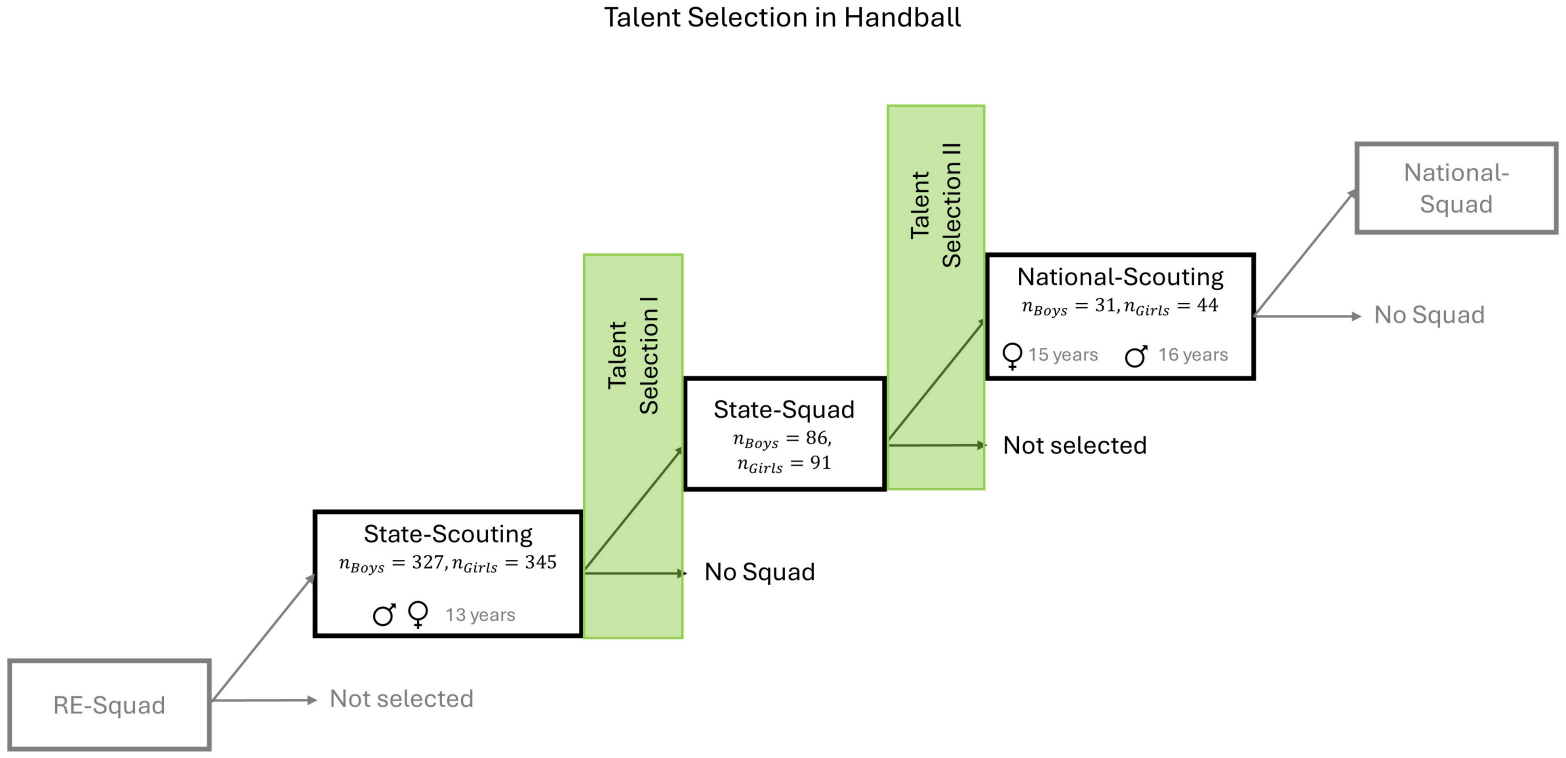
Path diagram of talent selection structures in the sport of handball. The period examined by the study is shown in black (the green markings indicate the two talent selection decisions).

Talent identification systems in handball are divided into several stages corresponding to different age groups. Athletes are selected for each stage accordingly. In Germany, talent selection is carried out by district associations, state associations and, based on this, by the national association. At the first stage, regional selections (RE-Squad) are formed at district level based on recommendations from clubs. This is followed by an initial systematic talent selection process carried out by the federal state association (State-Scouting). At the age of 13, athletes take part in motor performance tests as part of the selection process for the state-squad. In addition, the athletes' biological maturity is measured. The selected athletes are accepted into the state association's talent development structures (State-Squad) and receive additional support measures. At the age of 16 for boys and 15 for girls, members of the state-squad take part in the selection process of the national association (National scouting). The regional association selects 12 players per age group and gender for this selection process. The national association then makes a further selection from these athletes (National-Squad). Figure 1 illustrates the talent selection process in handball.

### 2.2 Study design and participants

A quantitative exploratory field study was conducted to investigate the research questions. Two selection decisions were analyzed for this purpose: talent selection I and talent selection II (see Figure 1). The quality criteria of an exploratory study by Ditroilo et al. (2025) were taken into account. The study features a large sample size and rigorous data collection (see Section 2.3).

The study was conducted as part of the annual state squad selection (State-Scouting) of the Hessian Handball Association between 2017 and 2023. A total of *N* = 967 (*N*_*Girls*_ = 524, *N*_*Boys*_ = 443) athletes took part in the selection process. The data analysis only includes athletes for whom all test results are available. Furthermore, the data analysis was carried out exclusively with the data of the field players, as the requirements profile of goalkeepers differs from that of field players (Krüger et al., 2016). The final sample size is *n* = 679 (*n*_*Girls*_ = 348, *n*_*Boys*_ = 331). The mean age of the girls was 12.93 ± 0.29 and that of the boys was 12.96 ± 0.19. On the one hand, the squad nomination at state level (talent selection I) is used as an indicator of performance. This nomination is made 3 months after the state squad selection by the Hessian Handball Association. On the other hand, the selection decision of the state association as to which athletes participate in National-Scouting (talent selection II) is used. This selection takes place 2.5 years after the state squad selection for boys and 1.5 years after the state squad selection for girls.

The athletes voluntarily participated in the motor performance testing and maturity assessment. They were free to withdraw from the selection process at any time. The athletes were informed in advance about the voluntary nature of their participation and their right to withdraw. This study was approved by the Ethics Committee of Department 02 at Johannes Gutenberg University Mainz (Decl. No.: 2025-12). Any investigation is in accordance with the Declaration of Helsinki 1964.

### 2.3 Measures

As part of the Hessian Handball Association's state squad selection process, the following six motor skills tests were conducted and maturation was determined by measuring anthropometric values. The tests were carried out in accordance with the guidelines of the Deutscher Handballbund (2024). The tests were carried out by students from Johannes Gutenberg University Mainz. The test supervisors were trained in advance in the use of the measuring instruments. At the start of the tests, all athletes were given a 30-min warm-up under the guidance of their respective regional coaches. After the warm-up phase, the athletes underwent the tests (sports motor skills tests and anthropometry) in a fixed order (rotation principle). Sufficient breaks were ensured between each test station to rule out fatigue effects. Each test station was supervised by two to three test supervisors.

#### 2.3.1 Sprint test (ST)

A 20-m sprint test without a starting signal was chosen to measure sprinting ability (Bös, 2023). The athletes were asked to run through two light barriers (Witty, Microgate) at maximum speed. Two attempts were made with a 60-s passive recovery. The athletes were to start in the high start position at a mark set up 30 cm in front of the first light barrier. Electronic timing starts when the player crosses the start line and stops when they cross the finish line. During the run, additional split times are recorded after 5 m and 10 m (Deutscher Handballbund, 2024). The best 20 m time was used for further analysis. Based on two test trials per athlete, a retest reliability of *r* = 0.90 was determined.

#### 2.3.2 Counter-movement jump (CMJ)

The counter-movement jump was used to measure vertical jumping power. The Optojump measuring instrument (Microgate, USA) was used to measure jump height. Three attempts were made with a 60-s passive recovery period. Prior to the test, the athletes were instructed in the jumping technique: stand upright with feet shoulder-width apart, arms fixed at the hips, swing back by bending the legs (knee angle approx. 90°) without pausing in this position, and land on both feet. The athletes were instructed to jump as high as possible. The highest jump value was used for further analysis. Based on two test trials per athlete, a retest reliability of *r* = 0.99 was determined.

#### 2.3.3 Shuttle run (SR)

The 20-m shuttle run test was used to measure aerobic and anaerobic endurance capacity (Léger and Lambert, 1982). The athletes are instructed to run back and forth as long as possible on a 20-meter track between two parallel lines, touching one of the lines each time an acoustic signal sounds. The frequency of the signals increases so that the running speed is increased by 0.5 km/h every minute from an initial speed of 8 km/h. At the start of the test, the athlete stands at a minimum distance of 1 m on one line. They start running when the first signal sounds. During the test, athletes are instructed to slow down or speed up to the next line if they arrive at the 20 m mark before or after the acoustic signal. The test ends when the athlete gives up or fails to touch the 20 m line (with at least one foot) twice in a row. In this case, the test supervisor ends the test. The test has a retest reliability of *r* = 0.89 (Léger et al., 1988).

#### 2.3.4 Push-up test (PT)

The push-up test was performed to measure upper body strength and endurance (Baumgartner et al., 2002). In the starting position, the female athletes' feet are on the floor and the male athletes' feet are on a gym bench. The athletes were asked to perform as many push-ups as possible. Before the test was performed, the athletes were instructed on the correct execution of the movement: in the starting position, the arms are extended, the hands are placed slightly wider than shoulder-width apart on the gym bench; in the end position, the chest touches the gym bench by bending the arms, and the starting position is reached by extending the arms. The speed of movement for performing the flexion or extension is 1 s and must be set by a metronome. One movement cycle therefore lasts 2 s (Deutscher Handballbund, 2024).

#### 2.3.5 Throwing test (TT)

The throwing test was performed to measure the speed in the throwing action in handball. The athletes were instructed to throw at maximum speed from a distance of 7 m at a centrally placed target area in the goal. A speed measurement device (V-MAXX) was set up behind the target area at a distance of 1.5 m behind the goal. Each athlete made two attempts with a 60-s passive recovery. The best speed result was used for further analysis. Based on two test trials per athlete, a retest reliability of *r* = 0.82 was determined.

#### 2.3.6 Dribbling test (DB)

The dribbling test measures the athletes' agility with the ball. The athletes were asked to dribble as fast as possible through a 3 × 5 m running course (in the shape of a figure eight) marked by five slalom poles. They were to start in the high start position. Electronic timing starts when the player crosses the starting line (Witty, Microgate). The running time is stopped when the player crosses the finish line again after the third run (Pabst et al., 2010). Based on two test trials per athlete, a retest reliability of *r* = 0.64 was determined.

#### 2.3.7 Maturity offset (M)

The maturity offset was determined using the Mirwald test based on the athlete's date of birth (age), standing height (height), sitting height, leg length (difference between the standing and sitting height), and weight. A mobile stadiometer (ADE, Hamburg, Germany) was used to determine height and sitting height. Digital scales were used to measure the athletes' weight to the nearest 0.1 kg. The maturity offset enables an estimation of the time interval (years) to the individual growth peak (PHV) and thus serves as an indicator of the biological maturity of children and adolescents. Negative maturity offset values indicate that the peak of growth is still ahead, while positive values indicate that it has already been exceeded. The following equation was used to determine the maturity offset (Mirwald et al., 2002):


$$\begin{array}{l} \text{For Boys}:9.236+0.0002708^{*}\left(Leg\;Length^{*}Sitting\;Height\right)\\ \;-0.001663^{*}\left(Age^{*}Leg\;Length\right)\\ \;+\;0.007216^{*}\left(Age^{*}Sitting\;Height\right)\\ \;+\;0.02292^{*}\left(\frac{Weight}{Height}\;\right) \end{array}$$

$$\begin{array}{l} \text{For Girls}:9.376\;+\;0.0001882^{*}\left(Leg\;Length^{*}Sitting\;Height\right)\\ \;+\;0.0022^{*}\left(Age^{*}Leg\;Length\right)\\ \;+\;0.005841^{*}\left(Age^{*}Sitting\;Height\right)\;0.002658^{*}\\ \;x\left(Age^{*}Weight\right)+\;0.07693^{*}\left(\frac{Weight}{Height}\;\right) \end{array}$$

### 2.4 Statistical analysis

#### 2.4.1 Variable-centered approach

A binary logistic regression model was used for the variable centered approach. The procedure was carried out separately according to gender and squad nomination level (State-Squad and National-Scouting; see Figure 1). To provide an overview of the probability of a player being nominated, the odds ratio (OR) was calculated as the effect size, with all statistical tests conducted at a significance level of *p* = 0 .05. Following Chen et al. (2010), OR values around 1.52 can be considered small effects, around 2.74 medium effects, and 4.72 or higher large effects. To examine the explanatory power of the logistic model, Nagelkerke's-*R*^2^ was analyzed (Backhaus et al., 2021; Höner et al., 2017). Data analysis was performed using SPSS 29.0.2.0 (IBM). Due to the exploratory research design, the sample size could not be determined a priori (Ditroilo et al., 2025). To determine which effect sizes are detectable with the given sample, a sensitivity power analysis was performed using G^*^Power (version 3.1.9.7, Heinrich Heine University Düsseldorf). The analysis yielded the following results: For talent selection I, the odds ratios were *OR*_*Boys*_ = 1.54 and *OR*_*Girls*_ = 1.51; for talent selection II, they were *OR*_*Boys*_ = 1.77 and *OR*_*Girls*_ = 1.82.

#### 2.4.2 Person-centered approach

This study uses cluster analysis as a person-centered method to group athletes according to similarities in motor performance and anthropometric traits (Howard and Hoffman, 2018; Morin et al., 2018). The cluster analysis involves the following steps: (1) First, hierarchical cluster analysis using the single linkage method is used to identify outliers; (2) These outliers were removed because they can distort the distance calculation and thus the cluster formation (to compare the variable-oriented and person-centered approaches, the identified outliers were also removed in the logistic regression); (3) Another hierarchical cluster analysis using the Ward method (squared Euclidean distance) is performed with the reduced data set; (4) The optimal number of clusters was determined using content-related (interpretability) and statistical criteria (dendrogram, elbow criterion and Mojena test with a threshold value of 2.75); (5) The cluster solution found in the hierarchical cluster analysis was optimized using a non-hierarchical cluster analysis (k-means method); (6) The split-half method was used to check the quality and robustness of the final cluster solution. Cohen's kappa coefficient was used as a measure of effect size (Backhaus et al., 2021; Bortz and Schuster, 2010; Landis and Koch, 1977). To determine the correlation between the individual clusters and performance in the form of squad selection, the ORs were calculated (Fahrmeir et al., 2023). Data analysis was performed using SPSS 29.0.2.0 (IBM).

## 3 Results

### 3.1 Variable-centered approach

Descriptive statistics from all tests are shown in Table 1.

**Table 1:** Descriptive statistic from all motor-performance and anthropometric variables for both genders.

Variable	Talent selection I				Talent selection II			
	Selected		Non-selected		Selected		Non-selected	
	*n*	*M* ± *SD*	*n*	*M* ± *SD*	*n*	*M* ± *SD*	*n*	*M* ± *SD*
Boys								
Sprint test (km/h)	86	6.03 ± 0.30	241	5.86 ± 0.30	31	6.07 ± 0.23	55	6.01 ± 0.33
Dribbling test (km/h)		2.13 ± 0.09		2.08 ± 0.10		2.13 ± 0.10		2.13 ± 0.09
Throwing test (km/h)		71.38 ± 6.16		66.43 ± 5.80		73.65 ± 5.53		70.11 ± 6.17
Counter-movement jump (cm)		40.68 ± 53.41		37.09 ± 52.76		40.63 ± 7.03		40.7 ± 7.52
Shuttle run (sec)		606.55 ± 84.26		560.55 ± 90.39		608.84 ± 94.87		605.25 ± 78.56
Push-up test (n)		23.7 ± 6.17		20.79 ± 6.59		23.87 ± 7.51		23.6 ± 5.34
Maturity offset (years)		0.02 ±.64		−0.39 ± 0.66		0.21 ±.54		0.08 ± 0.67
Girls								
Sprint test (km/h)	91	5.76 ± 0.23	254	5.66 ± 0.26	44	5.73 ± 0.24	47	5.78 ± 0.23
Dribbling test (km/h)		2.04 ± 0.09		1.97 ± 0.10		2.03 ± 0.10		2.04 ± 0.09
Throwing test (km/h)		65.29 ± 4.87		59.89 ± 5.30		66.27 ± 5.37		64.36 ± 4.19
Counter-movement jump (cm)		33.64 ± 5.87		32.85 ± 5.63		34.83 ± 6.28		32.53 ± 5.29
Shuttle run (sec)		496.82 ± 79.90		466.84 ± 83.74		508.63 ± 82.58		485.74 ± 76.52
Push-up test (n)		22.22 ± 8.12		19.72 ± 7.65		23.14 ± 8.39		21.36 ± 7.85
Maturity offset (years)		1.42 ± 0.43		1.24 ± 0.50		1.43 ± 0.48		1.4 ± 0.39

**Figure 2:**
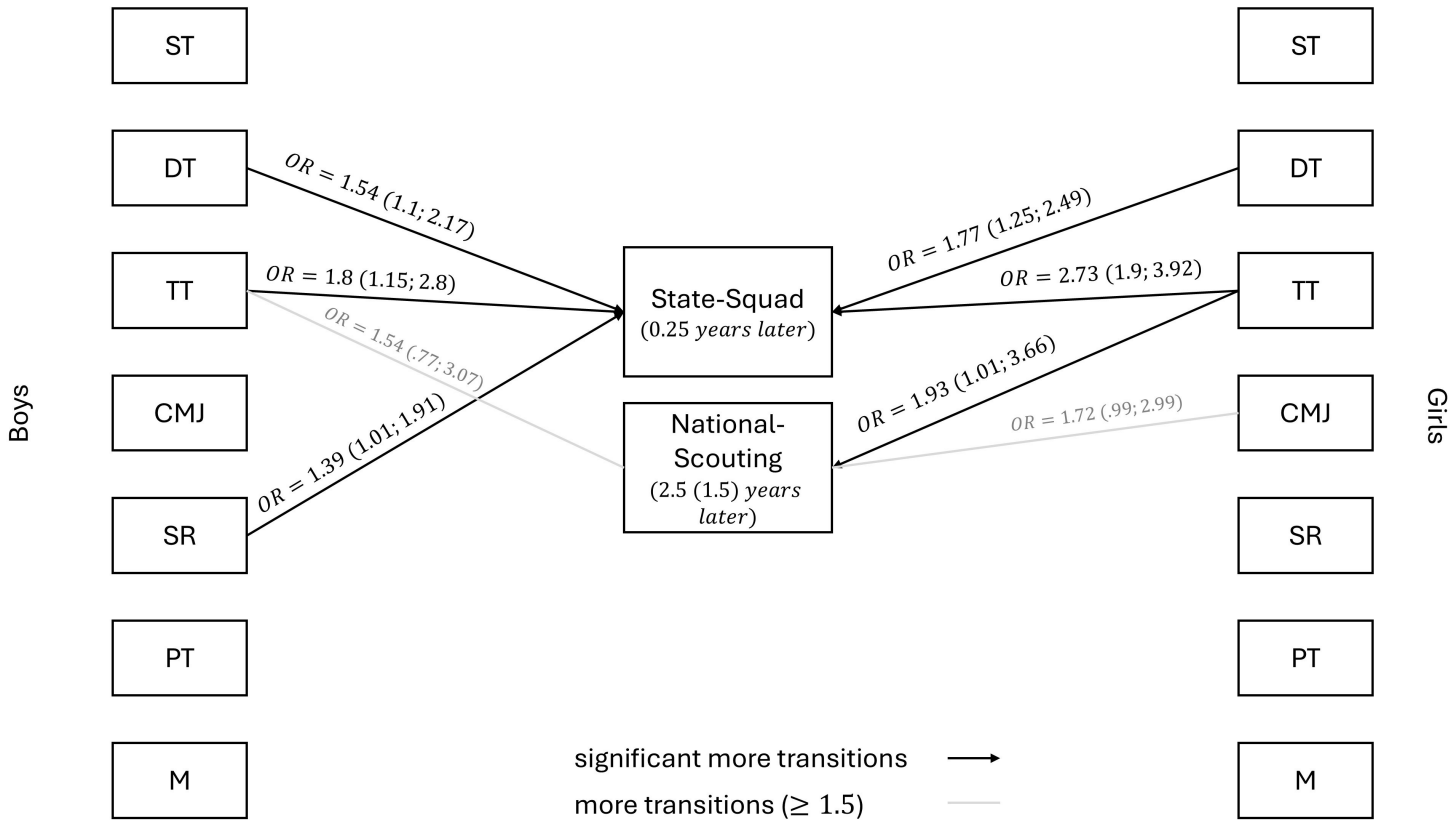
Odds ratio (OR) for the test results to illustrate the link between test results and squad selection (significant more transitions: OR > 1.0 with *p* < 0.05; more transition: *OR* > 1.0 *with p* > 0.05). Operating factors: ST, Sprint Test; DT, Dribbling Test; TT, Throwing Test; CMJ, Counter-movement Jump; SR, Shuttle-Run; PT, Push-Up Test; M, Maturity.

Table 2 and Figure 2 summarizes the influence of the individual test results on the respective selection decision, broken down by gender. Only those tests with an OR exceeding 1.5 are shown.

**Table 2:** Binary logistic regression for boys and girls.

Gender	Variable	B (SE)	Wald	*d*f	*P*	OR (CI)
Talent selection I						
Boys	Dribbling test	0.43 (0.17)	6.25	1	0.012	1.54 (1.10;2.17)
	Throwing test	0.58 (0.23)	6.69	1	0.010	1.80 (1.15;2.80)
	Shuttle run	0.33 (0.16)	4.08	1	0.043	1.39 (1.01;1.91)
Girls	Dribbling test	0.57 (0.18)	10.50	1	0.001	1.77 (1.25;2.49)
	Throwing test	1.01 (0.18)	29.72	1	<0.001	2.73 (1.90;3.92)
Talent selection II						
Boys	Throwing test	0.43 (0.35)	1.50	1	0.221	1.54 (1.01;1.91)
Girls	Throwing test	0.66 (0.33)	3.97	1	0.046	1.93 (1.01;3.66)
	Counter-movement jump	0.54 (0.28)	3.64	1	0.056	1.71 (0.99;2.99)

#### 3.1.1 Talent selection I

The analysis of the boys' binary logistic regression model for predicting talent selection I was able to classify 79.2% correctly and yielded a Nagelkerke-*R*^2^ of 0.262. This shows that 26.2% of the variance in State-Squad nomination among boys can be explained. In this model, the highest OR of 1.80 ([1.15;2.80], *p* < 0.05) was found for the throwing test.

In predicting talent selection I for female athletes, the logistic regression model achieved a classification accuracy of 78.6% and explained 32.4% of the variance in State-Squad nominations, as indicated by a Nagelkerke-*R*^2^ of 0.324. Among the motor tests included, the throwing test exhibited the strongest predictive effect 2.73 ([1.90;3.92], *p* < 0.05), while the dribbling test demonstrated a statistically significant positive association with squad nomination (OR = 1.77 [1.25;2.49], *p* < 0.05).

#### 3.1.2 Talent Selection II

Among boys, the logistic regression model achieved 64.8% classification accuracy for talent selection II (Nagelkerke-*R*^2^ = 0.0095; explaining 9.5% of variance in National-Squad nominations). None of the motor-skill tests showed a statistically significant association with invitations to the national scouting program. A non-significant positive correlation was found for the throwing test (OR = 0 1.54 [0.77; 3.07], *p* > 0.05).

For the female cohort, the logistic regression model predicting talent selection II correctly classified 69.2% of cases and explained 18.8% of the variance in National-Squad nominations (Nagelkerke-*R*^2^ = 0 .188). The throwing test emerged as the strongest predictor, yielding the highest odds ratio 1.93 ([1.01;3.66], *p* < 0.05). The throwing test demonstrates significant predictive power across both talent selection I and II, indicating its consistent relevance in later-stage selection. While a positive link was found for the dribbling test in the context of talent selection I, this relationship does not occur in talent selection II.

### 3.2 Person-centered approach

Against the backdrop of cluster analysis, the identification of outliers led to the removal of four cases among boys and three among girls from the data set. The descriptive statistics of the cluster solution found are presented in Table 3, differentiated by gender.

**Table 3:** Descriptive statistics (mean and standard deviation) of the operating factors.

	Sprint test (km/h)	Dribbling test (km/h)	Throwing test (km/h)	Jump and reach (cm)	Shuttle run (sec)	Push-up test (n)	Maturity offset (years)
	*M* ± *SD*	*M* ± *SD*	*M* ± *SD*	*M* ± *SD*	*M* ± *SD*	*M* ± *SD*	*M* ± *SD*
Boys							
Cluster throwing early maturing boys (*n* = 96)	5.82 ± 0.19	2.05 ± 0.08	69.19 ± 3.44	36.15 ± 5.93	530.21 ± 76.40	20.2 ± 5.45	0.04 ± 0.44
Cluster high performer early maturing boys (*n* = 100)	6.2 ± 0.20	2.14 ± 0.08	73.37 ± 4.97	43.74 ± 6.50	625.94 ± 73.63	24.3 ± 5.56	0.21 ± 0.44
Cluster low performer late maturing boys (*n* = 79)	5.57 ± 0.23	2.04 ± 0.08	61.99 ± 4.02	32.69 ± 6.46	523.59 ± 85.17	16.58 ± 6.32	0.89 ± 0.46
Cluster good performer late maturing boys (*n* = 52)	5.97 ± 0.18	2.19 00 ± 0.08	62.92 ± 4.00	38.66 ± 5.17	623.04 ± 71.73	26.33 ± 4.73	0.92 ± 0.48
Total (*n* = 327)	5.9 +0 .31	2.09 ± 0.10	67.73 ± 6.28	38.03 ± 7.43	572.65 ± 90.98	21.55 ± 6.60	− 0.28 ± 0.68
Girls							
Cluster jumper on-time maturing girls (*n* = 38)	5.76+.21	1.90 ±.08	59.29 ± 4.74	41.11 ± 2.94	480.32 ± 77.26	19.26 ± 5.85	1.30 ±0 .35
Cluster high performer on-time maturing girls (*n* = 72)	5.96+.16	2.09 ±.08	65.24 ± 4.76	37.40 ± 5.33	515.43 ± 69.03	26.24 ± 6.68	1.30 ± 0.41
Cluster endurance-power on-time maturing girls (*n* = 86)	5.64 ± 0.19	2.01 ± 0.07	60.42 ± 4.60	30.34 ± 3.58	530.0 ± 65.66	25.07 ± 6.12	1.26 ± 0.37
Cluster throwing early maturing girls (*n* = 81)	5.68 ± 0.19	2.01 ± 0.08	64.63 ± 4.15	31.21 ± 3.72	426.37 ± 63.52	14.58 ± 4.93	1.55 ±0 .36
Cluster low performer late maturing girls (*n* = 68)	5.41 ± 0.20	1.88 ±.08	55.46 ± 3.90	29.57 ± 3.99	416.32 ± 72.87	15.78 ± 6.73	1.0 ±0 .69
Total (*n* = 345)	5.68 ± 0 .26	1.99 ± 0 .11	61.31 ± 5.70	33.06 ± 5.70	474.75 ± 83.68	20.38 ± 7.85	1.29 ± 0.48

A four-cluster solution for boys and a five-cluster solution for girls were found. The application of the split-half method yielded moderate agreement (κ_*Boys*_ = 0.59; κ_*Girls*_ = 0.45) between the cluster solutions in both partial data sets separated by boys and girls.

**Figure 3:**
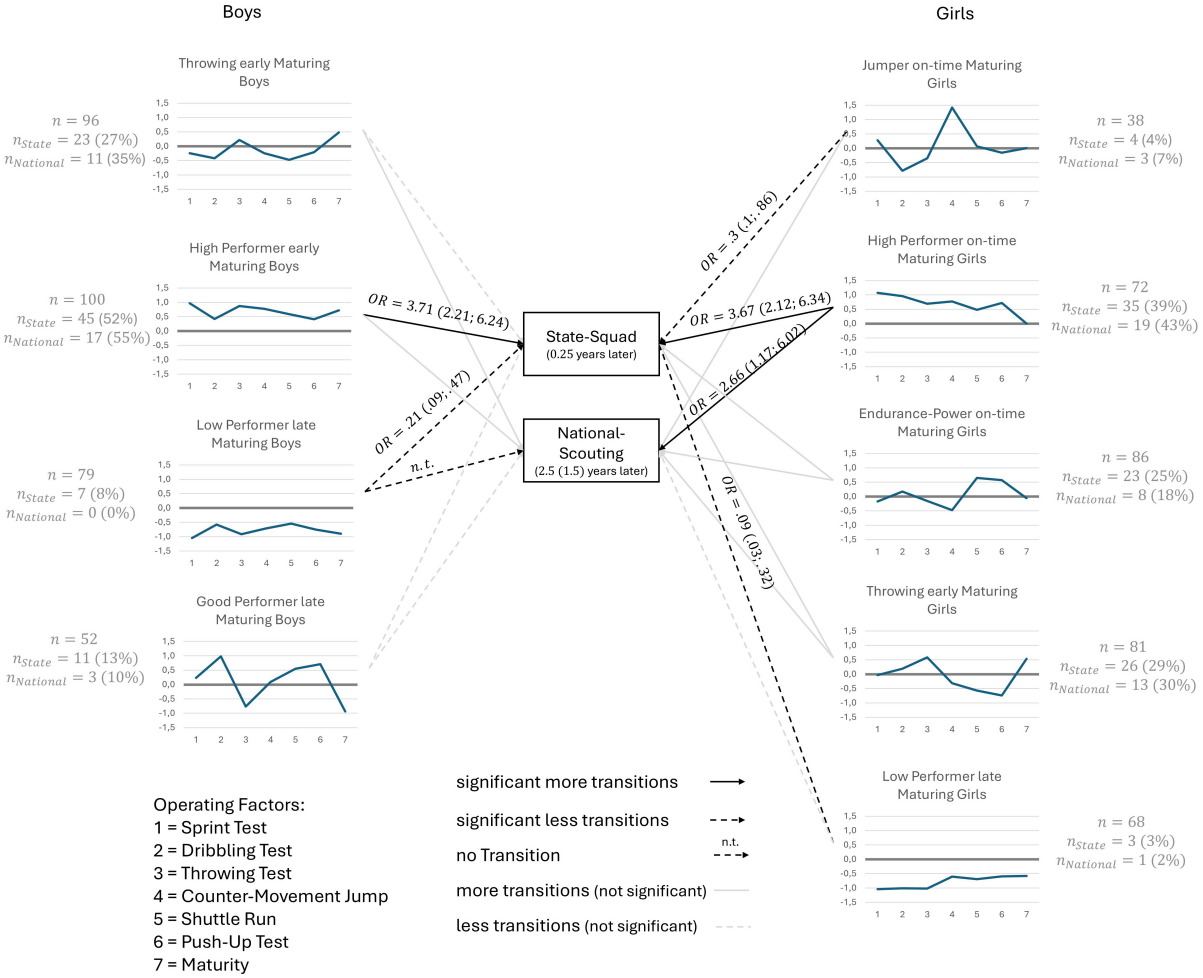
Profiles of z-scores of the 4-clusters for the boys and the 5-clusters for the girls and transitions to the performance levels. The numbers next to the arrows represent the odds rations (OR) and 95% CI (significant more transitions: OR > 1.0 with *p* > 0.05; significant less transitions: OR <1.0 with *p* > 0.05; more transition: OR > 1.0 with *p* > 0.05; less transitions: OR <1.0 with *p* > 0.05).

Figure 3 shows the respective mean values of the sports motor tests for the clusters, differentiated by gender as z-standardized values. Regardless of gender, the two contrasting clusters showed above-average [High Performer early Maturing Boys (*HPeMB*), High Performer on-time Maturing Girls (HPoMG)] and below-average [Low Performer late Maturing Boys (LPlMB), Late Performer late Maturing Girls (LPlMG)] performance in all tests. While the *HPeMB* cluster among boys comprises early-developing athletes in particular, the girls in the *HPoMG* cluster are average-developing athletes. The *LPlMB* and *LPlMG* clusters comprise late-developing athletes across both genders. In the sample of male athletes, the Throwing early Maturing Boys cluster (TeMB) was identified. This cluster comprises early-maturing athletes in particular who achieve below-average performance in all test results except the throwing test. In contrast to this cluster, the Good Performer late Maturing Boys cluster (GPlMB) was identified. This cluster comprises late-developing athletes in particular who achieve above-average performance in all test results except for the throwing test. The Jumper on-time Maturing Girls cluster (JoMG) was identified in the sample of female athletes. Athletes in this cluster are characterized by an average biological age and well above-average performance in the jump and reach test. Athletes with average development and high test results in the shuttle run and push-up test form the Endurance-Power on-time Maturing Girls cluster (EPoMG). Similar to the *TeMB* cluster for boys, the Throwing early Maturing Girls cluster (TeMG) comprises early-maturing athletes with above-average test results in the throwing test. The athletes achieved below-average scores in the sprint test, jump and reach, shuttle run and push-up test. The *TeMG* cluster for girls differs from the *TeMB* cluster for boys only in that the girls achieved slightly above-average scores in the dribbling test.

#### 3.2.1 Talent selection I

The OR was calculated to examine the link between the clusters and talent selection decision (see Figure 3). A significant link was found for appointment to the talent selection I. The *HPeMB* and *HPoMG* clusters were nominated with a higher probability compared to the other clusters (OR_*HPeMB*_ = 3.71, [2.21; 6.24]; OR_*HPeMG*_ = 3.67, [2.12; 6.34]). This shows that the clusters that are particularly frequently observed do not differ between boys and girls. Athletes in the *LPlMB and LPlMG* clusters were significantly less likely to be nominated to the State-Squad (OR_LPLMB_ = 0.21 [0.09; 0.47]; OR_LPlMG_ = 0.09, [0.03; 0.32]). In addition, it was found that female athletes in cluster *JoMG* were significantly less likely to be nominated for the State-Squad (OR = 0.30, [0.10; 0.86]).

#### 3.2.2 Talent selection II

In the context of talent selection II, no significant link could be identified for any cluster among the boys. It was found that no athletes from the *LPlMB* cluster participated in National-Scouting. Among girls, a significant link was found between the *HPoMG* cluster and National-Scouting. This cluster was invited to National-Scouting with the highest probability (OR = 2.66 [1.17; 6.02]). Athletes from the *LPlMG* cluster were invited to National-Scouting significantly less often (OR = 0.09 [0.03; 0.32]). It can be observed that among girls, the HPoMG cluster for talent selection I and talent selection II stands out as the profile that is nominated particularly frequently.

## 4 Discussion

The present exploratory study aims to (1) investigate the influence of athletic performance and maturation on selection decisions across different stages of the talent selection process, and (2) compare the explanatory power of variable-centered and person-centered approaches across different stages of the talent selection process. Specifically, the study examines which individual tests predict selection (variable-centered) and identifies player profiles based on motor skills and maturation to determine which profiles are more likely to be selected (person-centered), with all analyses differentiated by gender.

Similar to the results from soccer (Höner et al., 2017), the present study also showed that technical skills (dribbling and throwing tests) are key selection criteria in handball. A significant link was found among boys between the dribbling test (OR = 1.54) and throwing test (OR = 1.8) and talent selection I. For the girls, there was a significant correlation between the dribbling test (OR = 1.77) and the throwing test (OR = 2.73) and the squad nomination for talent selection I. In addition, the throwing test (OR = 1.77) was identified as a predictor for the girls in the context of talent selection II. The throwing test can be highlighted here, as it is a key selection criterion in talent selection I and II across both genders. This result is consistent with the findings of Koopmann et al. (2022). In addition to technical skills, a positive correlation between the shuttle run and the selection decision (talent selection I) was demonstrated for girls (OR = 1.39). This highlights the gender-specific differences in selection criteria, as identified in 3**×**3 basketball (Schmitz et al., 2024). Nevertheless, it should be noted that the current ORs for the dribbling test and throwing test (boys) are largely in the low range (OR = 1.52) according to Chen et al. (2010), and the shuttle run is below the threshold value. Only the throwing test is in the medium range (OR = 2.74).

Based on the person-centered approach, four clusters were identified for boys and five for girls. Across both genders, clusters of high performers (above-average performance in all tests) and low performers (below-average performance in all tests) were identified. The *TeMB* cluster was identified among the boys. This cluster is characterized by a high proportion of early-developing athletes and above-average performance in the throwing test. Athletes in this cluster achieved below-average results in the other motor skills tests. Similar to this cluster, the TeMG cluster was identified among girls. As with the boys, this cluster includes early-developing athletes who achieved above-average performance in the throwing test and below-average performance in the sprint, jump-and-reach, shuttle run and push-up tests. The TeMG cluster of girls differs from the TeMB cluster of boys in that the girls in the cluster achieved slightly above-average results in the dribbling test. The other clusters identified differ between the genders. In addition, the relationship between motor profiles and squad selection was analyzed. It was shown that boys and girls in the high performer cluster are most likely to be nominated in Talent Selection I (*OR*_*HPeMB*_ = 3.71, *R*_*HPoMG*_ = 3.67). Among the girls, a significant positive link was also found between high performers and talent selection II (*OR*_*HPoMG*_ = 2.66). The OR for Talent Selection I exceed the threshold value of OR = 2.74 specified by Chen et al. (2010). Therefore, this can be considered a medium to large effect. Similar to the findings of Zibung et al. (2016), the present study also shows that athletes with above-average performance in all tests, but without top scores in individual disciplines, are more frequently selected. For example, the boys in the *GPlMB* cluster achieve higher scores in the dribbling test than those in the *HPeMB* cluster.

In contrast to previous studies, which predominantly consider one research approach in isolation, the present study systematically compares the variable-centered and person-centered approaches. The complementary consideration of the two approaches shows that the results complement each other in their significance. For example, the variable-centered approach leads to the finding that athletes with above-average results in the throwing test are significantly more likely (OR_Boys_ = 1.8, OR_Girls_ = 2.73) nominated for the State-Squad (talent selection I). Beyond the isolated consideration of this single test result, the person-centered approach shows that athletes with above-average performance across all motor skills tests are significantly more likely to be nominated for the State-Squad (OR_*HPeMB*_ = 3.71, OR_HP*oMG*_ = 3.67). This underscores the role of motor skills as a central component of the performance structure model of sports games, in which they interact with other factors such as tactical skills (Weigel, 2014). Superior motor skills can promote the expression and development of these other components, thereby improving overall athletic performance. This assumption is supported by findings from research on the 'eye of the coach, which examines what coaches look for when identifying talent. Here, too, motor skills are often highlighted as a key assessment factor (Roberts et al., 2019). In addition, Hohmann (2001) describes that performance conspicuousness is a performance diagnostic criterion of athletic talent. Against this background, the profile of high performers can be interpreted as an expression of such a criterion.

In addition, based on the variable-centered approach's finding that the throwing test is a significant predictor of squad nomination, the other clusters can be better classified. For example, girls in the *TeMG* cluster are nominated for the State-Squad more often (OR = 1.47 [0.84;2.5]) than girls in the other clusters. Athletes in this cluster are characterized by above-average performance in the throwing test. In comparison, athletes in the *JoMG* cluster have slightly below-average scores in this test. Athletes in this cluster are significantly less likely to be nominated for the State-Squad (OR = 0.30). A comparison of the ORs of these two clusters supports the importance of the throwing test as determined by the variable-centered approach.

For sports practice, this finding underscores the current relevance of the throwing test in talent scouting. At the same time, the person-centered approach shows that clusters characterized by above-average performance in this test contain a high proportion of early-developing athletes. Similarly, the High Performer profile among boys is also distinguished by a high degree of early maturation, indicating that advanced biological development contributes substantially to top-level performance within this group (La Rubia et al., 2024). This findings may indicate a selection bias favoring early-maturing athletes, a phenomenon that has been documented and investigated repeatedly within talent research. It further underscores the critical influence of biological maturation on motor performance, suggesting that differences in developmental timing can affect current performance outcomes (La Rubia et al., 2024; Massa et al., 2022; Matthys et al., 2011; Thieschäfer et al., 2025). This highlights the need to consider maturation status when interpreting motor test results and making talent selection decisions, to avoid overlooking late-developing athletes with high long-term potential. Potential selection biases (e.g., favoring early-developing athletes) in talent selection I have a significant impact on talent selection II, as only athletes from the State-Squad can participate in National-Scouting. This leads to the assumption that sampling bias already occurs at the time of talent selection II. Therefore, the results of this study emphasize that coaches must take the biological maturity of athletes into account in their selection decisions.

The limitations of this study are outlined below. The period between talent selection I and talent selection II is 3.5 years for boys (and 2.5 years for girls; see Figure 1). Due to the cross-sectional study design, the study cannot make any statements about the athletes‘ development over this period. At the same time, various influencing factors (e.g., environmental conditions, biological development) have an impact on the athletes' development during this period. These are directly associated with performance. Due to the exclusion of goalkeepers from the sample, no conclusions can be drawn regarding the goalkeeper position in relation to the selection criteria. Furthermore, the study only collects motor data, meaning that other dimensions of top performance are not taken into account.

### 4.1 Conclusions and future research

In light of the research aim (1), which examined the impact of motor performance and biological maturation on talent selection, the present study demonstrates that relevant selection criteria partially shift between Talent Selection I and II across both variable-centered and person-centered approaches. For example, among girls, dribbling and throwing tests were most predictive at talent selection I, whereas Counter-Movement jump and Throwing tests were more relevant at talent selection II. These findings highlight that the relative importance of individual performance factors changes over the course of athlete development, suggesting that different tests may need to be prioritized at different stages of the selection process. As these results stem from an exploratory study, all potential links identified in this paper need to be confirmed in a future study with adequate error control (confirmatory research). In addition, future research should examine the temporal stability and evolution of the explanatory power of talent factors across multiple selection decisions. Investigating how the predictive relevance of individual performance components changes over successive stages of the talent identification process would provide valuable insights into the dynamics of athlete development and help optimize the timing and design of assessment protocols.

With regard to the research aim (2), the isolated consideration of individual motor skills in the variable-centered approach demonstrates only limited predictive power for squad nomination, due to small to moderate effect sizes (OR ≤ 2.73). In contrast, the person-centered approach provides higher prognostic validity, as it accounts for the interaction of multiple performance factors. This enhanced predictive power can be observed consistently across both selection stages. These findings highlight the relevance of performance profile analysis for TID systems. At the same time, further research is needed to determine how future profiling approaches can be designed to create more holistic profiles that integrate further dimensions of top performance (e.g., psychological abilities, sport-specific skills and environmental factors).

## Data Availability

The raw data supporting the conclusions of this article will be made available by the authors, without undue reservation.
